# Time-tuning cancer therapy

**DOI:** 10.18632/aging.100793

**Published:** 2015-08-20

**Authors:** Gustavo Baldassarre, Ilenia Segatto, Barbara Belletti

**Affiliations:** Division of Experimental Oncology 2, Department of Translational Research, C.R.O. Aviano IRCCS, National Cancer Institute, 33081 Aviano, Italy

Personalized medicine in cancer is based on targeted therapy, often defined as the right drug to the right patient. Decades of research aimed to establish the appropriate targets for each cancer subtype have led to enormous advances in patients' treatment. This is particularly evident in breast cancer (BC) patients, especially HER2+ and HR+ ones, for whom a panel of targeted therapeutic options are today available.

These advances, together with implementation of screening/prevention programs, have resulted in significant decline in BC mortality and augmented detection of early lesions. In early BC, when tumor cells are not supposed to have activated their metastatic program yet, the main therapeutic objective resides in restraining local recurrence [1].

Following surgery, clinically undetected clusters of neoplastic cells may remain either locally or at distant sites and may eventually develop into clinically detectable recurrence. As long as the microenvironment surrounding these tumors provides tumor-suppressive signals, these masses will not progress. For these neoplastic clusters to grow into frank cancer a subversion of tissue homeostasis must occur and signals from the microenvironment need to awake BC cells. Surgery itself may undoubtedly represent one of such subverting factors and, indeed, it has been proposed that it may impact on the growth kinetics of BC micro metastasis [2]. Consistently, post-surgical inflammation and subsequent wound healing process lead to massive release of cytokines and growth factors and inflammation-induced signals are known to activate positive feedback loops that allow the maintenance of a transformed state even when the triggering event is no longer present [3].

These clinical and molecular observations have prompted us to the research of novel peri-surgical treatments, aimed to killing residual tumor cells by affecting their crosstalk with the microenvironment right in the moment in which these cells might have to choose between life (or even dormancy) and death. To this aim, we started to investigate the properties of surgical wound fluids (WF) in the breast cancer context. These WF are drained from BC patients, for 24 hours after surgery, and can be used as a surrogate model of the wound response in vitro, since they contain all stimuli produced in the breast microenvironment during post-surgical responses [4–6]. We have evaluated the signaling pathways preponderantly activated by WF, using a large panel of BC cell lines. Strong and specific activation of the STAT3 pathway was consistently observed [7].

The role of STAT3 in promoting transformation is well documented [3], yet the merit of our work was to link for the first time STAT3 activation to breast cancer relapse after surgery. Constitutive activity of STAT3 has been observed in 35% to 60% of human breast tumors and in many BC cell lines. Moreover, STAT3 activity has been implicated in the regulation of the stem-like properties of tumor initiating cells (TICs). These cells have the inherent ability to generate tumors exploiting stem-cell processes, such as self-renewal and many evidences indicate that they may account for drug- and radio-resistance and for disease relapse and metastasis formation. We observed that WF potently stimulates the cancer initiating phenotypes and self-renewal of BC cells. Genetic and/or pharmacological inhibition of STAT3 completely prevented the acquisition of such phenotypes, suggesting that the inflammatory stimuli present in the post-surgical setting in breast microenvironment enhanced the stem-like properties of TICs, at least in part, via STAT3 activation. IL-6 triggers many of the STAT3-related phenotypes. It is interesting to note that the use of the IL-6 blocking antibody in the presence of WF also impacted on self-renewal of BC cells, but failed to induce a suppression comparable to that obtained with STAT3 inhibition, suggesting that although IL-6 is present and active in the WF [4], it did not represent the principal mediator of STAT3 activation in this setting nor was it the only/primary cytokine mediating the WF ability to stimulate stem-like phenotypes in BC cells. Using a mouse model of breast cancer recurrence [5], well recapitulating the course of the human disease, we could confirm in vivo the relevance of STAT3 pathway in the occurrence of BC relapse following surgery.

Our findings suggest that STAT3-dependent cellular changes may allow the BC cells to overcome anti-tumorigenic pressures from the microenvironment. In this context, STAT3 may be considered a promising target but, for its inhibition to result really successful, the correct definition of the target tumor population and the identification of the best time window for treatment will be critical.

Research for novel cancer therapies has largely relied on proliferation as an endpoint. However, targeting the crosstalk between cancer cells and components of the microenvironment is likely to provide much more profound clinical benefits. Surgery elicits a range of inflammatory- and wound healing-responses that could provide a sort of ‘start signal’ for the survival and/or awakening of residual cancer cells. Our studies suggest that choosing not only the right drug for the right patient, but also the right time to administer treatments, i.e. the peri-surgical setting, may more efficaciously interrupt the dialogue between the inflammatory stroma and the residual breast cancer cells. This approach may result crucial for the fate of these cancer cells as well as for cure of the patient.
